# Designing Mobile Phone Text Messages Using the Behavior Change Wheel Framework to Influence Food Literacy in Adults With Type 2 Diabetes in Kenya: Protocol for a Systematic Development Study

**DOI:** 10.2196/48271

**Published:** 2023-12-04

**Authors:** Moses Mokaya, Florence Kyallo, Peter Yiga, Janna Lena Koole, Tessy Boedt, Roman Vangoitsenhoven, Christophe Matthys

**Affiliations:** 1 Department of Human Nutrition Sciences Jomo Kenyatta University of Agriculture and Technology Nairobi Kenya; 2 Experimental and Clinical Endocrinology Department of Chronic Diseases and Metabolism KU Leuven Leuven Belgium; 3 Mildmay Research Centre Kampala Uganda; 4 Department of Endocrinology University Hospitals Leuven Belgium

**Keywords:** behavior change techniques, Behavior Change Wheel, type 2 diabetes, low-income populations, mHealth, mobile health, glycemic control, adults, diabetes, Africa, mobile phone, support care, care, support, behavior, diabetes

## Abstract

**Background:**

The worldwide prevalence of type 2 diabetes (T2D) has increased in the past decade, and it is projected to increase by 126% by 2045 in Africa. At the same time, mobile phone use has increased in Africa, providing a potential for innovative mobile health interventions to support diabetes care.

**Objective:**

This study aimed to apply the Behavior Change Wheel (BCW) framework to develop text messages to influence food literacy in adults with T2D in urban Kenya.

**Methods:**

The 8 steps of the BCW framework guided the development of text messages: (1) Define the problem in behavioral terms; (2) select target behaviors; (3) specify the target behaviors based on who needs to perform the behaviors, what needs to change, and when, where, how often, and with whom; (4) identify what needs to change; (5) identify intervention functions; (6) select policy categories; (7) select behavior change techniques (BCTs); and (8) select the mode of delivery. Recent exploratory studies in Kenya and other low- and middle-income countries provided information that was used to contextualize the intervention.

**Results:**

In step 1, the behavioral problem was defined as unhealthy dietary patterns among adults with T2D. In step 2, based on a qualitative study in the target population, the target behavior was selected to be evaluation of reliable sources of information, and selection and preparation of healthy food. In step 3, unhealthy dietary patterns were selected. In step 4, 10 domains of the Theoretical Domains Framework were identified, and in step 5, 5 intervention functions were linked to the domains and unhealthy dietary patterns were specified. In step 6, communication and regulations were identified as policy categories, while in step 7, 9 BCTs were selected from the Behavior Change Technique Taxonomy version 1. In step 8, the most suitable mode of delivery was determined to be mobile text messages. A total of 36 mobile text messages were developed based on the 9 BCTs.

**Conclusions:**

This study shows the step-by-step application of the BCW framework to develop mobile text messages to influence food literacy in adults with T2D.

**International Registered Report Identifier (IRRID):**

RR1-10.2196/48271

## Introduction

Worldwide, the prevalence of diabetes has been on the rise in the past decade, with more than half a billion people with diabetes in 2021, of which more than 90% had type 2 diabetes (T2D) [1]. Currently, in Africa, 24 million people have diabetes, and it is projected that this prevalence will rise by 126% to 55 million in 2045 [1]. This projected rise in the African region will be the highest compared to all other worldwide regions [1]. In Kenya, 3% of adults aged 20-79 years have diabetes, with a higher prevalence in urban (3.4%) compared to rural (1.9%) areas [2]. Further, in Kenya, older age (60-69 years) is associated with a higher incidence of diabetes [2]. In addition to the rising prevalence of T2D in Kenya, only 36.6% of patients achieve glycemic control [3]. Glycemic control in diabetes is defined as glycated hemoglobin (HbA_1c_) levels of <7% (53 mmol/mol) [4]. Poor glycemic control increases the risk of the development and progression of micro- and macrovascular complications in people with diabetes [5]. As such, achieving glycemic control soon after diagnosis is an important goal in the management of diabetes. In addition to diabetes self-management medical actions, a recent prospective analysis showed that optimization of dietary patterns is an effective strategy to achieve glycemic control [6]. Poor dietary quality has been assumed to be associated with a lack of food-related knowledge and skills [7]. However, food literacy has been used to improve dietary behavior associated with healthy dietary patterns [8,9]. Food literacy enables the application of information about food choices and critical reflection on the effect of food choices and has the potential to prevent disease, promote optimal health, and sustain the environment [9,10].

As the prevalence of T2D is on the rise, mobile phone subscription in low- and middle-income countries (LMICs) has been on the rise in the past 2 decades. Recent data show that mobile cellular subscriptions per 100 people in LMICs and Kenya rose from nearly 0% in 2000 to 104% and 123%, respectively, in 2021 [11]. This increase in mobile phone subscriptions in LMICs has resulted in a commensurate rise in the use of mobile health (mHealth) in behavior change interventions [12,13]. Optimization of glycemic control in adults with T2D through a food literacy intervention requires behavior change. Therefore, a systematic approach to developing an intervention and its subsequent implementation and evaluation was used [14]. In this study, we used the Behavior Change Wheel (BCW) framework [15] to systematically develop intervention components. Given that the BCW framework has been used widely to design and evaluate several behavior change interventions [16-20], we used it to develop text messages. Specifically, the BCW framework helps in the systematic identification of target behaviors that need to be changed to improve health outcomes and to select appropriate intervention functions, policy categories, and behavior change techniques (BCTs) based on the analysis of the sources of behavior (capability, opportunity, and motivation) [14].

**Figure 1:**
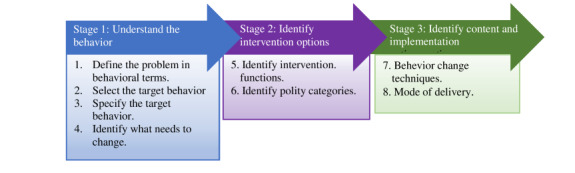
Stages and steps in the BCW. Adapted from Michie et al [15]. BCW: Behavior Change Wheel.

**Figure 2:**
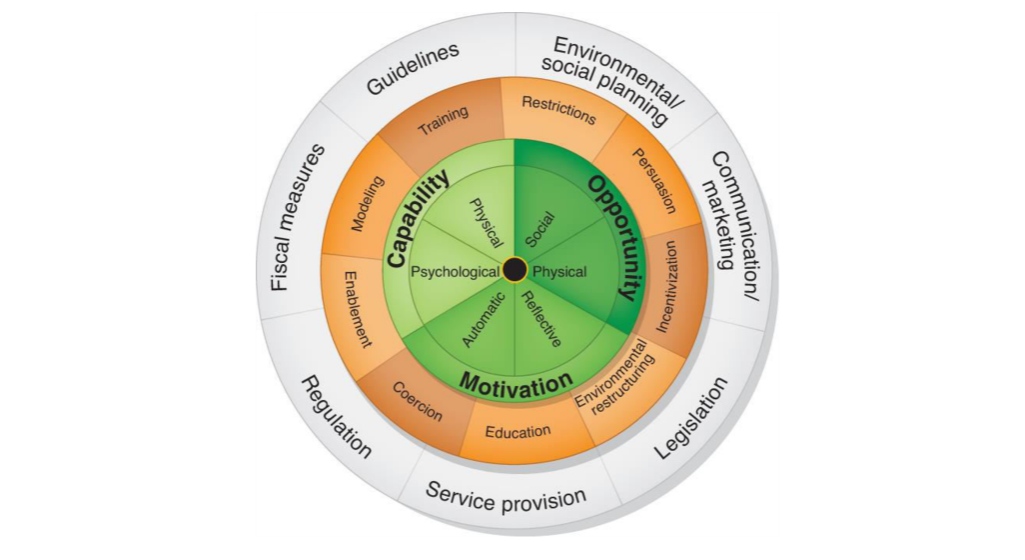
The COM-B model. Adapted from Michie et al [15]. COM-B: capability, opportunity, motivation for behavior.

The BCW is a 3-stage comprehensive framework for designing complex interventions (Figure 1) that integrates behavior theory by using expert consensus and a validation process [14]. The BCW framework has been designed to help understand and select relevant mechanisms of action for an intervention [15]. The BCW framework uses the 3-layered capability, opportunity, motivation for behavior (COM-B) model (Figure 2) to analyze and diagnose behavior. The first, core layer of the COM-B model is further expanded to physical and psychological capability, social and physical opportunity, and automatic and reflective motivation. According to Michie et al [15], core domains are needed to increase the likelihood of performing the target behavior. COM-B is supported by the Theoretical Domains Framework (TDF), which describes 14 constructs from 33 behavior change theories [21]. The second layer of the BCW framework is composed of 9 intervention functions: education, persuasion, incentivization, coercion, training, enablement, modeling, environmental restructuring, and restrictions. These intervention functions indicate how an intervention changes behavior and are linked to a taxonomy of 93 replicable BCTs (Behavior Change Technique Taxonomy version 1 [BCTTv1]) [22]. The third, outermost layer of the BCW framework is composed of 7 policy categories that can be applied to support the delivery of the intervention functions. Although text messaging is increasingly used in health care and the demand for interventions based on theory, existing text messaging interventions often overlook the theoretical basis for their development [23,24]. This study therefore aimed to apply the BCW framework to develop text messages to influence food literacy in adults with T2D in urban Kenya. The specific objectives of this study were to (1) define the problem affecting adults with T2D in behavioral terms, (2) define the intervention and implementation options of the identified target behaviors, and (3) develop text messages to influence food literacy in adults with T2D in Kenya.

## Methods

### Study Design

Developing this intervention involved 3 stages of the BCW framework: (1) understanding the behavior and user preferences, (2) identifying intervention options, and (3) identifying content and implementation options. These stages were further subdivided into 8 steps and are further explained in detail later (Figure 1). The process of intervention development was iterative and was conducted by the research team (authors MM, FK, PY, RV, TB, JLK and CM). The team consisted of dieticians, nutritionists, public health specialists, and an endocrinologist.

#### Stage 1: Understanding the Behavior and User Preferences

##### Step 1: Define the Problem in Behavioral Terms

Defining the problem in specific behavioral terms included an in-depth description of the target population and the specific behavior [15]. We used the recent and contextually relevant literature [25-27] to refine our understanding of the target population’s dietary behavior to optimize glycemic control from health providers’ and patients’ viewpoints.

##### Step 2: Select Target Behaviors

Target behaviors were selected by applying a systems analysis approach, as recommended by Michie et al [15]. First, we used the list of facilitators of and barriers to healthy dietary behavior in adults with TDM from our previous phenomenological qualitative study conducted in Kenya [28]. In that study, 30 male and female respondents were interviewed through mobile telephones, revealing that facilitators of and barriers to healthy dietary behavior are related to food literacy. We ranked the various facilitators and barriers based on the number of thematic nodes (collection of references about a specific theme) generated by NVivo software (Lumivero). Table 1 shows the ranking of thematic nodes.

**Table 1:** Ranking of thematic nodes.

Food literacy component	Number of nodes	Rank of priority
Plan	42	4
Prepare	60	3
Select	168	2
Eat	171	1

Second, we used the criteria recommended by BCW guidelines to prioritize the target behaviors. The criteria include the following considerations: (1) the relative impact of the behavior, (2) the likelihood of changing the behavior, (3) the potential for spilling over into other relevant dietary behaviors, and (4) the ease of measurement of the behavior. Finally, we categorized each selected behavior as very promising, promising, unpromising but worth considering, or unacceptable based on guidance by Michie et al [15]. These decisions were made by consensus by the expert panel and then tabulated for each selected behavior.

##### Step 3: Specify the Target Behavior

Step 3 involved specifying the context in which the target behavior will occur by asking specific questions: Who needs to perform the behavior? What needs to be done differently to achieve the change? Where and when do they need to perform the behavior? How often and with whom did they perform the behavior? To answer each of these questions, we used the findings from our qualitative study conducted on the target population [28].

##### Step 4: Identify What Needs to Change

Step 4 involved 2 substeps: (1) behavioral analysis using the COM-B model and (2) identification of what needs to change using the TDF.

In substep 1 of behavioral analysis, we mapped the specified target behavior from step 3 to the COM-B component and further explored what needed to happen for the target behavior to occur and whether there was a need for change to select and eat food known to contribute to glycemic control.

In substep 2, after determining what needs to change, we linked the behavior that needed to change based on BCW and TDF guidelines [15,29]. The TDF consists of 14 domains: knowledge; skills; memory, attention, and decision processes; behavioral regulation; social/professional role and identity; beliefs about capabilities; optimism; beliefs about consequences; intentions; goals; reinforcement; environment context and resources; social influences; and emotion. The domains identified in this step were tabulated and summarized to include the following aspects for the specified behavior: (1) COM-B component, (2) what needs to happen for the target behavior to occur, (3) whether there is a need for behavior change, (4) the domain linked to the COM-B component, and (5) relevance of the domain.

#### Stage 2: Identifying Intervention Options

##### Step 5: Identify Intervention Functions

Intervention functions were mapped onto each of the theoretical domains identified in step 4 [15]. The intervention functions that would most likely affect behavior change were selected based on the COM-B and TDF behavior analyses conducted in step 4. The relevant intervention functions were then assessed using APEASE (acceptability, practicability, effectiveness, affordability, side effects, and equity) criteria of the BCW framework [15]. The assessment included checking how the selected intervention functions meet the 5 components of the APEASE criteria.

##### Step 6: Select Policy Categories

The policy categories included communication/marketing, guidelines, fiscal measures, regulation, legislation, environmental/social planning, and service provision, which guide decisions made by authorities that help support and enact interventions [15]. We mapped the policy categories onto the intervention functions identified in step 5 using the APEASE criteria [15]. The selection of relevant policy categories was based on the matrix of links between intervention functions and policy categories described by Michie et al [15] and the APEASE criteria. The decisions on the APEASE criteria were informed by our understanding of the context, as revealed by our qualitative study [28].

#### Stage 3: Identifying Content and Implementation Options

##### Step 7: Identify BCTs

Based on the intervention functions identified in step 5, we selected BCTs from the BCTTv1 [22] and the APEASE criteria. The BCTTv1 is a standardized terminology used to specify the active ingredients of behavior change interventions and consists of 93 unique BCTs [22].

##### Step 8: Select the Mode of Delivery

The mode of delivery was guided by the taxonomy of the models of delivery for intervention functions that involved communication. Based on the taxonomy of the models of delivery, we selected the mode of delivery by using the findings from our qualitative study [15,30]. The taxonomy is structured into binary options, where we selected the most practical option using our understanding of the context and the target population [28].

### Testing the Feasibility of the Intervention

The BCW guidelines recommend that developed behavior change interventions be tested for feasibility. As such, we were guided by the Medical Research Council (MRC) framework that illustrates the steps to be followed in the development and evaluation of behavior change interventions [31].

### Ethical Considerations

This being a study on the development of text messages to enhance food literacy in adults with T2D, human subjects were not directly involved. However, studies [25,27,28,30] that provided evidence in the development of this study sought ethical approval.

## Results

### Summary of Findings

**Figure 3:**
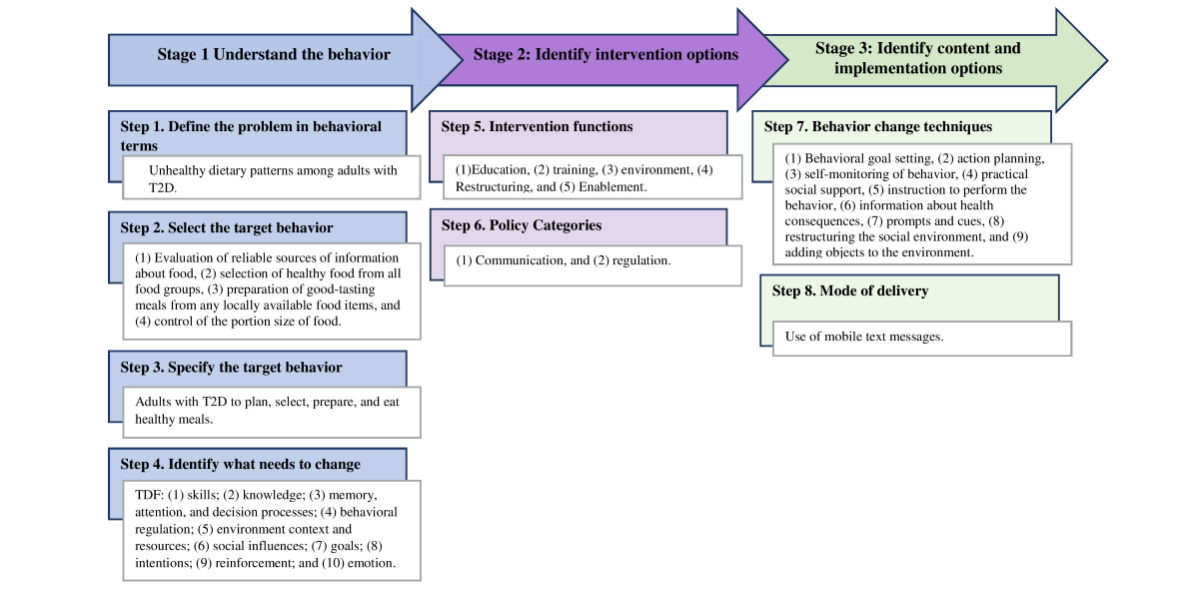
Summary of findings from the steps in the Behavior Change Wheel.

Figure 3 summarizes findings from the 8 steps of the BCW framework.

#### Stage 1: Understanding the Behavior and User Preferences

##### Step 1: Define the Problem in Behavioral Terms

In defining the problem in behavioral terms, we considered findings from existing evidence in LMICs [25,27] and our previous studies [28,30]. The existing literature, however, is limited by the fact that it was conducted in different settings and that most of these studies lacked the rigor for replication in other settings [26]. Our qualitative study of facilitators of and barriers to healthy dietary behaviors among adults with T2D revealed the following facilitators: knowledge of healthy food choices, gardening, self-efficacy, food preparation skills and eating at home, education by health care workers, food availability, proximity to food-selling points, and family support. The barriers included tastes and preferences of food, health conditions barring the intake of certain foods, random eating of unhealthy food, socioeconomic status, seasonal unavailability of fruits, food safety concerns, and inaccurate beliefs and information about food and diet. In a systematic review of self-management of diabetes in sub-Saharan Africa, which included 43 studies, most of which were observational, there were misconceptions about and gaps of knowledge in what entails healthy food [27]. Further, the review found that interventions on dietary behavior led to relevant improvements in healthy eating habits. Another systematic review of adherence to diabetes self-care behaviors in LMICs found that adherence to dietary recommendations ranged from 29.9% to 91.7%, while consumption of fruits and vegetables averaged 3 times per week, although the portion sizes were not revealed [25]. In summary, the key gaps identified in these studies were related to our qualitative study [28], showing that dietary behavior is associated with food literacy. Food literacy is “the interrelated combination of knowledge, skills and self-efficacy on planning for meals, selecting foods, preparing food, eating and evaluating information about food with the ultimate goal of developing a lifelong healthy, sustainable and gastronomic relationship with food” [32]. Food literacy enables the application of information about food choices and critical reflection on the effect of food choices and has the potential to prevent disease, promote optimal health, and sustain the environment [9,10]. As such, the fundamental components of food literacy can be applied to positively influence behaviors required for healthy diet patterns. In practical terms, food literacy comprises planning, selection, preparation, and eating of food [33]. Based on these findings, we defined the problem in behavioral terms as unhealthy dietary patterns among adults with T2D.

##### Step 2: Select Target Behaviors

Based on a system analysis, we selected the following as target behaviors: evaluation of reliable sources of information about food, selection of healthy food from all food groups, preparation of good-tasting meals from any locally available food items, and control of the portion size of food (Table 2).

Based on the findings of our qualitative study on the same population [28], we prioritized the target behaviors in Table 3.

**Table 2:** Identification of target behaviors.

Facilitator/barrier	Contextualized target behavior (components of food literacy) [33]
Access to reliable sources of information about food	Evaluation of information
Access to healthy food from all food groups	Selection of healthy food
Preparation of good-tasting meals from any locally available food items	Preparation of healthy meals
Control of the portion size of food	Eating a healthy diet

**Table 3:** Prioritization of the target behaviors.

Potential target behavior relevant to improving food literacy	Impact of behavior change^a^	Likelihood of changing behavior^b^	Spillover score^c^	Measurement score and means of measurement^d^
Evaluate reliable sources of information about food.	Very promising	Promising	Very promising	Promising (identify sources of reliable information)
Select healthy food from various sources to ensure varied consumption from all food groups.	Very promising	Promising	Very promising	Promising (proportion of healthy foods accessed)
Prepare healthy and good-tasting meals from locally available food and ingredients.	Promising	Unpromising but worth considering	Very promising	Promising (healthy and good-tasting meals prepared)
Consume a controlled portion size of food.	Promising	Promising	Promising	Promising (number of meals eaten that fit the plate model^e^)

^a^Likely impact if the behavior were to be changed.

^b^Ease of likelihood to change dietary patterns.

^c^Likelihood of having an impact on other behaviors that may support a change in dietary patterns.

^d^Measurability either by routine data or through new data collection procedures.

^e^Plate model: a visual method for teaching meal planning, where the dinner plate serves as a pie chart to illustrate the proportions of the plate that should be covered by various food groups [34].

##### Step 3: Specify the Target Behavior

The target behavior was specified by answering the following questions, as illustrated in Table 4:

- Who? (Adults with T2D)
- Where? (At home or work)
- When? (When planning, selecting, eating, or preparing food)
- How often? (Every time or most of the time during consumption)
- With whom? (Either alone or with the family)

**Table 4:** Specification of the target behavior.

Specification	Description
Target behavior	Adults with T2D^a^ to plan, select, prepare, and eat healthy meals
Who needs to perform the behavior?	Adults with T2D
What is to be done differently to achieve the desired change?	Plan, select, prepare, and eat healthy meals
When is it to be done?	Whenever eating
Where is it to be done?	At home, at work, or away from home
How often do they need to do it?	Every day
To whom do they need to do it?	Alone or with family or friends

^a^T2D: type 2 diabetes.

##### Step 4: Identify What Needs to Change

Identification of what needs to change was informed by findings drawn from the opinions in our qualitative study [28], in addition to recent and contextually relevant findings from Uganda [26]. Table 4 summarizes what needs to change based on the behavioral diagnosis using the COM-B model. A total of 10 TDF domains were identified: skills; knowledge; memory, attention, and decision processes; behavioral regulation; environmental context and resources; social influences; goals; intentions; reinforcement; and emotion. The behaviors diagnosed were further expanded to illustrate their relevance (Table 5).

**Table 5:** Behavioral analysis and application of the TDF^a^ in diagnosis.

COM-B^b^ component and what needs to happen for the target behavior to occur		Is there a need for change?		TDF domain linked to the COM-B component		Relevance of the domain
**Physical capability**						
	Search and access healthy food from all food groups.	Yes	Physical skills		Explain how to search for and access healthy food from all food groups.	
	Prepare healthy meals using locally available food ingredients.	Yes	Physical skills		Explain how to prepare healthy meals using locally available food ingredients.	
	Be able to serve controlled portion sizes using the plate model.	Yes	Physical skills		Explain how to serve controlled portion sizes using the plate model.	
**Psychological capability**						
	Know how to distinguish between reliable information and myths.	Yes	Knowledge		Create knowledge of various food groups and food sources.	
	Know where to search for reliable information.	Yes	Knowledge		Create knowledge on where to search for reliable information.	
	Know various food groups and locally available health food sources.	Yes	Knowledge		Create information on food groups and locally available food sources.	
	Advanced planning skills to ensure the availability of healthy food.	Yes	Memory, attention, and decision processes; behavioral regulation		Enable action-planning skills.	
	Know foods to avoid for optimal glycemic control.	Yes	Knowledge		Help in decision-making on the type and quality of healthy food.	
	Know the composition of the plate model.	Yes	Knowledge		Enable the serving of healthy portions of food.	
**Physical opportunity**						
	Be aware of where to get healthy foods (shops, markets, home garden), and establish or maintain kitchen gardens.	No: food is accessible in the target population.^c^	N/A^d^		N/A	
	Use the mobile phone or computer to search for information, where possible.	No: the proportion of the target population using smartphones or computers is low.^c^	N/A		N/A	
	Have the necessary cooking equipment.	No: patients commonly cook at home.^c^	N/A		N/A	
	Use smaller plates when serving food.	Yes	Environmental context and resources		Search and use a smaller plate to serve food.	
**Social opportunity**						
	Call health care workers when searching for information.	No: patients have access to health care workers during clinic visits.^c^	N/A		N/A	
	Challenge cultural beliefs regarding food choice.	Yes	Social influences		Understand that some cultural values are unhealthy.	
	Awareness to family members involved in meal preparation on the preparation of healthy meals.	Yes: some families provide support to adults with T2D.^a^	Social influences		Social support in the selection, preparation and eating of healthy food.	
	Change cultural habits regarding food portion size.	Yes	Social influences		Change perceptions on the amount of food to be eaten.	
**Reflective motivation**						
	Intend and prioritize seeking clarification of information when needed	Yes	Intentions		Encourage the intention to search for information through reliable sources.	
	Establish routines and habits to eat healthy meals	Yes	Goals		Develop routines to eat healthy meals.	
	Intend to purchase and eat healthy food	Yes	Intentions		Encourage the intention to purchase and eat healthy food.	
	Plan to cook healthy meals	Yes: The target population cooks most meals at home.^c^	Goals		Plan to cook healthy meals.	
	Plan to serve healthy portion sizes	Yes	Goals (action planning)		Enable service of healthy portion sizes.	
**Automatic motivation**						
	Desire to look for information when in doubt	Yes	Reinforcement		Reinforce the habit of searching for reliable information.	
	Establish meal plans to ensure the intake of healthy meals	Yes	Emotion		Plan for healthy meals.	
	Establish routines and habits of serving healthy portions of food	Yes	Reinforcement		Have the desire to serve healthy portions of food.	

^a^TDF: Theoretical Domains Framework.

^b^COM-B: capability, opportunity, motivation for behavior.

^c^Findings from our qualitative study [28].

^d^N/A: not applicable (because there is no need for change).

#### Stage 2: Identifying Intervention Options

##### Step 5: Identify Intervention Functions

Based on the APEASE criteria, a total of 4 intervention functions were mapped onto the 10 TDF domains identified in step 4. The identified intervention functions included education, training, environment restructuring, and enablement (Table 6).

**Table 6:** Mapping intervention functions to corresponding COM-B^a^ components and BCTs^b^.

COM-B component and TDF^c^ domain linked to it		Intervention function	Selected BCT
**Physical capability**			
	Physical skills	Train how to search for and access healthy food from all food groups.	Instruction to perform the behavior
	Physical skills	Train how to prepare healthy meals using locally available food ingredients.	Instruction to perform the behavior
	Physical skills	Train how to serve controlled portion sizes using the plate model.	Instruction to perform the behavior
**Psychological capability**			
	Knowledge	Educate where to search for reliable information.	Information about health consequences
	Knowledge	Educate on various food groups.	Information about health consequences
	Knowledge	Educate food local foods in various food groups.	Information about health consequences
	Knowledge	Educate about food groups and locally available health food sources.	Information about health consequences
	Knowledge	Educate foods to avoid for optimal glycemic control.	Information about health consequences
	Knowledge	Train on the composition of the plate model.	Instruction to perform the behavior
	Behavioral regulation	Train or enable planning for and accessing healthy food.	Instruction to perform the behavior
	Memory, attention, and decision processes	Enable the distinction between reliable information and myths.	Information about health consequences
	Memory, attention, and decision processes	Enable decision-making on the type and quality of healthy food.	Action planning
**Physical opportunity**			
	Environmental context and resources	Enable environmental restructuring to use a smaller plate to serve meals.	Adding objects to the environment
**Social opportunity**			
	Social influences	Enable environmental restructuring to modify cultural beliefs on food that affect the choice of food.	Restructuring the social environment
	Social influences	Enable to challenge sociocultural habits regarding food portion sizes.	Practical social support
	Social influences	Enable awareness creation among family members involved in meal preparation on how to prepare healthy meals.	Practical social support
**Reflective motivation**			
	Intentions	Educate how to clarify information, when needed.	Prompts and cues
	Goals	Enable establishment routines and habits to eat healthy meals.	Behavioral goal setting
	Goals	Enable establishment routines and habits of observing optimal portion sizes when serving food.	Behavioral goal setting
	Goals	Enable planning to serve correct portion sizes.	Behavioral goal setting
	Goals	Enable planning to cook healthy meals.	Action planning
	Intentions	Educate to prioritize the purchase and eating of healthy food.	Self-monitoring of behavior
	Beliefs and consequences	Educate on foods to avoid for optimal glycemic control.	Information about health consequences
**Automatic motivation**			
	Reinforcement	Persuade to look for information when in doubt.	Practical social support
	Goals	Enable to establish meal plans to ensure the intake of healthy meals.	Practical social support

^a^COM-B: capability, opportunity, motivation for behavior.

^b^BCT: behavior change technique.

^c^TDF: Theoretical Domains Framework.

##### Step 6: Identify Policy Categories

Policy categories were selected based on our qualitative study after analysis using the APEASE criteria: (1) Most participants (90%) had an income of less than 400 euros (US $ 422.27) per month, and this had limited the control of fiscal decisions; (2) all participants owned a basic mobile phone; and (3) the process of developing guidelines by the Ministry of Health in Kenya is structured and involves multiple stakeholders, making it likely to be limited by time. Based on this, we identified communication and regulation as the most practical policy categories.

#### Stage 3: Identifying Content and Implementation Options

##### Step 7: Identify BCTs

BCTs are the “irreducible, observable, and replicable components of an intervention designed to redirect behaviour” [35]. In this study, we selected 9 BCTs from the taxonomy of behavior change [36] to develop intervention content aimed at influencing dietary behavior (Table 5). These BCTs included behavioral goal setting, action planning, self-monitoring of behavior, practical social support, instruction to perform the behavior, information about health consequences, prompts and cues, restructuring the social environment, and adding objects to the environment.

##### Step 8: Select the Mode of Delivery

Based on findings from our qualitative study [28], most of the adults with T2D in the target population owned and used mobile phones. Additionally, our systematic review revealed that using text messages in LMICs is associated with a clinically significant effect on HbA_1c_ levels, in addition to being cheap and easy to use irrespective of socioeconomic status, and is not affected by racial disparities [30,37,38]. As such, we selected mobile text messages as the mode of delivery [28].

**Figure 4:**
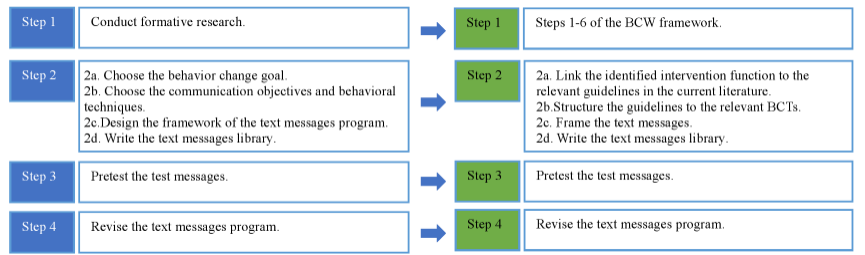
Adaptation of the message development. Adapted from Abroms et al [39].

The text messages were developed based on Abroms et al’s [39] 4 steps for developing a text messaging program: (1) formative research, (2) design, (3) pretest, and (4) revision. In this study, we modified steps 1 and 2, as shown in Figure 4. Specifically, we modified step 2 to include 3 substeps: (2a) linking the identified intervention function to relevant Kenyan and international guidelines and recommendations for a healthy diet [42**-**47], (2b) structuring guideline content to the relevant BCT, and (2c) gain- or loss-framing the text message. Gain-framed health promotion messages emphasize the benefits of engaging in a certain behavior, while a loss-framed message emphasizes the consequences of failing to participate in the behavior [46]. The messages were developed through a consultative process that included MM, CM, and FK. Table 7 illustrates a sample of the developed text messages.

**Table 7:** Sample text messages to be used in the mHealth^a^ intervention.

COM-B^b^ components and what needs to happen for the target behavior to occur		BCT^c^	Mobile text messages
**Physical capability**			
	Be able to select healthy food	Instructions on how to perform the behavior	To meet nutrient needs within calorie limits, choose a variety of nutrient-dense foods across and within all food groups in recommended amounts. Energy-dense foods include vegetables, fruits, whole grains, beans, nuts and seeds, and lean protein when prepared with little or no saturated fats, added sugars, and sodium. There are 5 food groups: (1) vegetables and legumes or beans; (2) fruit; (3) lean meats and poultry, fish, eggs, nuts and seeds, and legumes or beans; (4) grain (cereal) foods, mostly whole-grain or high-cereal fiber varieties; and (5) milk, yoghurt, cheese, or alternatives, mostly reduced fat. Most processed carbohydrate foods release glucose more quickly than whole-grain carbohydrates. Eat whole-grain carbohydrates, which produce a slower rise in blood glucose levels, which are called low-glycemic-index (GI) foods and result in better blood glucose control.
**Psychological capability**			
	Know healthy food.	Information about health consequences	The amount of carbohydrates in your meal has the greatest effect on blood glucose levels. Avoid big servings of carbohydrates in your meals and ensure most of your energy-giving foods are composed of whole meal or high fiber. Make vegetables and fruits take the larger portion of your plate. There are 2 main types of fat: saturated and unsaturated. Excessive saturated fat in foods, such as fatty meat, sausages, and butter, can increase the amount of cholesterol in the blood, which increases your risk of developing heart disease.
	Use advanced planning skills to ensure the selection of healthy food.	Information about health consequences	Select whole or minimally processed foods, which help control your blood glucose. Make an eating plan each week: this is the key to fast and easy meal preparation. This will also ensure that you plan for healthy diets and better-controlled blood glucose.
	Know the composition of the plate model.	Information about health consequences	Start with a 9-inch dinner plate. Fill half with nonstarchy vegetables, such as managu, Sukuma wiki, green beans, broccoli, cauliflower, cabbage, and carrots. Fill one-quarter with a lean protein, such as chicken, fish, legumes, beans, or eggs. Fill one quarter with whole-meal carbohydrate foods: grains, starchy vegetables (eg, potatoes and peas), rice, pasta, beans, fruit, and yoghurt. A cup of milk also counts as a carbohydrate food.
**Social opportunity**			
	Change cultural habits on the portion size.	Restructuring the physical environment	Controlled portion sizes of food are important to reduce calorific intake. To control your portion sizes, plan to use a smaller plate size when serving meals. Your health is a personal responsibility. When eating, try to avoid places that may entice you to eat excessive food. Eat most of your meals at home for better control of healthy food and your blood sugar.
**Reflective motivation**			
	Intend to select a healthy diet.	Information about health consequences, prompts/cues	Make your shopping list ahead of time and do not go shopping while hungry. This helps you buy healthier items but also saves money and helps you select healthier foods. Instead of purchasing processed grade 1 maize flour or Unga that is processed, use posho-milled Unga or whole-meal flour. Posho-milled flour contains fiber and other plant extracts that help in blood sugar control.
	Intend to prioritize eating a healthy diet.	Information about health consequences, prompts/cues	Small changes to more nutrient-dense, single food and beverage choices combine to make a nutrient-dense meal and can lead to a whole day of nutrient-dense meals and snacks, increasing your blood sugar control. Place fresh and locally available low-carbohydrate fruits on the table or a place that is easy to reach to increase intake of fruits. This will enable you to eat healthy and avoid unhealthy snacking.
**Automatic motivation**			
	Establish routines and habits to select a healthy diet.	Instructions on how to perform a behavior	Keep a food diary for a few days to evaluate what you eat every day. Note how you were feeling when you ate: hungry, not hungry, tired, or stressed? Create a list of cues by reviewing your food diary to become more aware of when you’re triggered to eat for reasons other than hunger. Is there anything else you can do to avoid the cue or situation? If you cannot avoid it, do something different that would be healthier. Replace unhealthy habits with new, healthy ones.
	Establish routines and habits to select a healthy diet.	Prompts/cues	Replace white rice with whole-grain foods or brown rice. This ensures better blood sugar control.
	Establish routines and habits to serve and eat a healthy diet.	Instructions on how to perform behavior	Eat smaller meals more often. Eat at least 3 meals a day, with snacks in between. When you wait too long to eat, you are more likely to make unhealthy food choices.

^a^mHealth: mobile health.

^b^COM-B: capabilities, opportunities, and motivation for behavior.

^c^BCT: behavior change technique.

### Testing the Feasibility of the Developed Intervention

According to the Medical Research Council (MRC) guidance on developing and evaluating complex interventions, the development of an intervention in this study is the first of 4 steps [31]. The other subsequent and iterative steps include feasibility, evaluation, and implementation. Based on this guidance, the intervention has been tested for feasibility through an exploratory trial in the target population, and the findings will be reported elsewhere.

## Discussion

### Principal Findings

This paper described the systematic development of an mHealth intervention using mobile text messages to optimize glycemic control in adults with T2D in Kenya following the BCW framework. Preparatory analysis preceding this study revealed that adults with T2D in Kenya have unhealthy dietary patterns and lack reliable sources of information to enhance food literacy [28]. The diagnosis also revealed that there are barriers to the target population’s capability, opportunity, and motivation relating to food literacy. Based on the diagnosis, 4 intervention functions and 9 BCTs were selected to promote food literacy. Specific BCTs were selected to be integrated into this intervention: for example, adding objects to the environment (eg, using a smaller plate to serve food for portion control) and prompts or cues (eg, replacing white rice with whole-meal foods). The identified mHealth mode of delivery was mobile text messaging based on the practical applicability in the older population of adults with T2D.

When designing this study, we identified food literacy as a channel to address the problem of poor glycemic control. Food literacy has the potential to facilitate dietary behavior change through the connection of food and nutrition-related knowledge, and cooking skills [47]. Considering that food literacy explicitly focuses on health literacy skills in a food context [48], a positive correlation between health literacy and diabetes knowledge has been reported in patients with T2D [49]. Overall, the appropriate application of food literacy is associated with positive health outcomes in adults with T2D.

In this study, we used the BCW framework and contextual evidence to develop text messages for adults with T2D. The approach of basing the development on theory and the content of the text messages on the needs of the target population has been used variedly in recent studies [50-53]. In a recent 2-country African study [50], the BCW framework was used to develop text messages in 4 phases that included exploration of primary and secondary data and focus group discussions (FGDs), which were followed by pretesting through telephone interviews. Bartlett et al [51] developed text messages for patients with diabetes in the United Kingdom based on BCTs in a process that included health care specialist workshops, FGDs, and acceptability and fidelity surveys. MacPherson et al [53] developed text messages guided by BCTs, the motivational interviewing counselling style, and the Small Steps for Big Changes approach [54]. However, unlike our study in which we specifically developed text messages to facilitate food literacy, these studies [50,51,53] have focused on the wider multicomponent approach to diabetes management and prevention. The focused approach in our study was based on evidence showing that food literacy is an important problem in the target population [28], while a multicomponent approach to diabetes care is effective in optimizing glycemic control [55,56].

### Strengths and Limitations

We acknowledge some strengths and weaknesses in using the BCW framework in this study. First, this study used contextual data to conduct behavioral diagnosis, which included our qualitative study of facilitators of and barriers to healthy dietary behavior in the target population, as recommended by Buchanan et al [57]. Second, the BCW framework provides a systematic method for designing a theory-based intervention, starting from a behavioral diagnosis of what needs to change, followed by linking the diagnosis to intervention functions, policy categories, and BCTs to change the target behavior. This approach enables the intervention to be contextualized to the needs of the target population, which increases its chances for success [58]. Third, the BCW framework uses a harmonized language of theoretical constructs and BCTs, which are important for the replication and synthesis of research and evidence [59,60].

However, this study was limited by the BCW framework’s lack of a structured framework for operationalizing BCTs into mHealth methods of delivery [61], leaving it open to the creativity of the intervention developers and the context of the target population. However, to address this limitation, we modified previous guidance on the development of text messages [39] and developed messages based on target behaviors following guidelines on healthy diets that are linked to BCTs.

### Conclusion

This study reported the systematic use of the BCW framework, the COM-B model, the TDF, and BCTs based on the contextual needs of the target population to develop text messages to influence food literacy in adults with T2D. The efficacy of the text messages will be evaluated through an exploratory trial in adults with T2D in the target population.

## Abbreviations

APEASE: acceptability, practicability, effectiveness, affordability, side effects, and equity
BCT: behavior change technique
BCTTv1: Behavior Change Technique Taxonomy version 1
BCW: Behavior Change Wheel
COM-B: capability, opportunity, motivation for behavior
FGD: focus group discussion
HbA_1c_: glycated hemoglobin
mHealth: mobile health
MRC: Medical Research Council
T2D: type 2 diabetes
TDF: Theoretical Domains Framework

## Data Availability

The data generated during this study are available from the corresponding author upon reasonable request.

## References

[1] (2021). IDF diabetes atlas tenth edition, 2021. International Diabetes Federation.

[2] Mohamed SF, Mwangi M, Mutua MK, Kibachio J, Hussein A, Ndegwa Z, Owondo S, Asiki G, Kyobutungi C (2018). Prevalence and factors associated with pre-diabetes and diabetes mellitus in Kenya: results from a national survey. BMC Public Health.

[3] Otieno FC, Mikhail T, Acharya K, Muga J, Ngugi N, Njenga E (2021). Suboptimal glycemic control and prevalence of diabetes-related complications in Kenyan population with diabetes: cohort analysis of the seventh wave of the International Diabetes Management Practices Study (IDMPS). Endocr Metab Sci.

[4] American Diabetes Association (2018). Standards of medical care in diabetes—2018 abridged for primary care providers. Clin Diabetes.

[5] Stratton IM, Adler AI, Neil HA, Matthews DR, Manley SE, Cull CA, Hadden D, Turner RC, Holman RR (2000). Association of glycaemia with macrovascular and microvascular complications of type 2 diabetes (UKPDS 35): prospective observational study. BMJ.

[6] Garbutt J, England C, Jones AG, Andrews RC, Salway R, Johnson L (2022). Is glycaemic control associated with dietary patterns independent of weight change in people newly diagnosed with type 2 diabetes? Prospective analysis of the Early-ACTivity-In-Diabetes trial. BMC Med.

[7] Spronk I, Kullen C, Burdon C, O'Connor H (2014). Relationship between nutrition knowledge and dietary intake. Br J Nutr.

[8] Cullen T, Hatch J, Martin W, Higgins JW, Sheppard R (2015). Food literacy: definition and framework for action. Can J Diet Pract Res.

[9] Krause C, Sommerhalder K, Beer-Borst S, Abel T (2018). Just a subtle difference? Findings from a systematic review on definitions of nutrition literacy and food literacy. Health Promot Int.

[10] Colatruglio S, Slater J, Deer F, Falkenberg T, McMillan B, Sims L (2014). Food literacy: bridging the gap between food, nutrition and well-being. Sustainable Well-Being: Concepts, Issues, and Educational Practices.

[11] Mobile cellular subscriptions (per 100 people). The World Bank.

[12] Afable A, Karingula NS (2016). Evidence based review of type 2 diabetes prevention and management in low and middle income countries. World J Diabetes.

[13] Beratarrechea A, Moyano D, Irazola V, Rubinstein A (2017). mHealth interventions to counter noncommunicable diseases in developing countries still an uncertain promise. Cardiol Clin.

[14] Michie S, van Stralen MM, West R (2011). The behaviour change wheel: a new method for characterising and designing behaviour change interventions. Implement Sci.

[15] Michie S, Atkins L, West R (2014). The Behaviour Change Wheel: A Guide To Designing Interventions.

[16] Walsh JC, Groarke JM (2019). Integrating behavioral science with mobile (mHealth) technology to optimize health behavior change interventions. Eur Psychol.

[17] Chiang N, Guo M, Amico KR, Atkins L, Lester RT (2018). Interactive two-way mHealth interventions for improving medication adherence: an evaluation using the Behaviour Change Wheel framework. JMIR Mhealth Uhealth.

[18] Nelligan RK, Hinman RS, Atkins L, Bennell KL (2019). A short message service intervention to support adherence to home-based strengthening exercise for people with knee osteoarthritis: intervention design applying the Behavior Change Wheel. JMIR Mhealth Uhealth.

[19] Mahdi S, Michalik-Denny EK, Buckland NJ (2022). An assessment of behavior change techniques in two versions of a dietary mobile application: the Change4Life food scanner. Front Public Health.

[20] Haley JA, Rhind DJA, Maidment DW (2023). Applying the Behaviour Change Wheel to assess the theoretical underpinning of a novel smartphone application to increase physical activity in adults with spinal cord injuries. Mhealth.

[21] Cane J, O'Connor D, Michie S (2012). Validation of the theoretical domains framework for use in behaviour change and implementation research. Implement Sci.

[22] Michie S, Richardson M, Johnston M, Abraham C, Francis J, Hardeman W, Eccles MP, Cane J, Wood CE (2013). The behavior change technique taxonomy (v1) of 93 hierarchically clustered techniques: building an international consensus for the reporting of behavior change interventions. Ann Behav Med.

[23] Sahin C, Courtney KL, Naylor PJ, E Rhodes R (2019). Tailored mobile text messaging interventions targeting type 2 diabetes self-management: a systematic review and a meta-analysis. Digit Health.

[24] Arambepola C, Ricci-Cabello I, Manikavasagam P, Roberts N, French DP, Farmer A (2016). The impact of automated brief messages promoting lifestyle changes delivered via mobile devices to people with type 2 diabetes: a systematic literature review and meta-analysis of controlled trials. J Med Internet Res.

[25] Mogre V, Johnson NA, Tzelepis F, Shaw JE, Paul C (2019). A systematic review of adherence to diabetes self-care behaviours: evidence from low- and middle-income countries. J Adv Nurs.

[26] Yiga P, Seghers J, Ogwok P, Matthys C (2020). Determinants of dietary and physical activity behaviours among women of reproductive age in urban sub-Saharan Africa: a systematic review. Br J Nutr.

[27] Stephani V, Opoku D, Beran D (2018). Self-management of diabetes in Sub-Saharan Africa: a systematic review. BMC Public Health.

[28] Mokaya M, Saruni E, Kyallo F, Vangoitsenhoven R, Matthys C (2022). Perceived facilitators and barriers to healthy dietary behaviour in adults with type 2 diabetes mellitus in Kenya: a qualitative study. Public Health Nutr.

[29] Atkins L, Francis J, Islam R, O'Connor D, Patey A, Ivers N, Foy R, Duncan EM, Colquhoun H, Grimshaw JM, Lawton R, Michie S (2017). A guide to using the Theoretical Domains Framework of behaviour change to investigate implementation problems. Implement Sci.

[30] Mokaya M, Kyallo F, Vangoitsenhoven R, Matthys C (2022). Clinical and patient-centered implementation outcomes of mHealth interventions for type 2 diabetes in low-and-middle income countries: a systematic review. Int J Behav Nutr Phys Act.

[31] Skivington K, Matthews L, Simpson SA, Craig P, Baird J, Blazeby JM, Boyd KA, Craig N, French DP, McIntosh E, Petticrew M, Rycroft-Malone J, White M, Moore L (2021). A new framework for developing and evaluating complex interventions: update of Medical Research Council guidance. BMJ.

[32] Boedt T, Voorend R, Derboven J, Dancet E, Spiessens C, Matthys C (2020). Development of a food literacy intervention for couples trying to conceive. Proc Nutr Soc.

[33] Vidgen HA, Gallegos D (2014). Defining food literacy and its components. Appetite.

[34] Camelon KM, Hådell K, Jämsén PT, Ketonen KJ, Kohtamäki HM, Mäkimatilla S, Törmälä ML, Valve RH (1998). The plate model: a visual method of teaching meal planning. J Am Diet Assoc.

[35] Michie S, Abraham C, Eccles MP, Francis JJ, Hardeman W, Johnston M (2011). Strengthening evaluation and implementation by specifying components of behaviour change interventions: a study protocol. Implement Sci.

[36] Kok G, Gottlieb NH, Peters GY, Mullen PD, Parcel GS, Ruiter RAC, Fernández ME, Markham C, Bartholomew LK (2016). A taxonomy of behaviour change methods: an intervention mapping approach. Health Psychol Rev.

[37] Duggan M (2013). Cell phone activities 2013. Pew Research Center.

[38] Chakkalakal RJ, Kripalani S, Schlundt DG, Elasy TA, Osborn CY (2014). Disparities in using technology to access health information: race versus health literacy. Diabetes Care.

[39] Abroms LC, Whittaker R, Free C, Mendel Van Alstyne J, Schindler-Ruwisch JM (2015). Developing and pretesting a text messaging program for health behavior change: recommended steps. JMIR Mhealth Uhealth.

[40] (2019). Dietary guidelines for the Belgian adult population, report no. 9284. Superior Health Council.

[41] Barness LA (1979). Dietary guidelines. Arch Pediatr Adolesc Med.

[42] Gonzalez Fischer C, Garnett T (2016). Plates, pyramids and planets. Developments in national healthy and sustainable dietary guidelines: a state of play assessment. Food and Agriculture Organization of the United Nations.

[43] National Health and Medical Research Council (2013). Australian Dietary Guidelines: Providing the Scientific Evidence for Healthier Australian Diets.

[44] (2015). 2015 – 2020 Dietary guidelines for Americans. 8th edition. U.S. Department of Health and Human Services and U.S. Department of Agriculture.

[45] (2017). National guidelines for healthy diets and physical activity. Ministry of Health.

[46] Rothman AJ, Salovey P (1997). Shaping perceptions to motivate healthy behavior: the role of message framing. Psychol Bull.

[47] Begley A, Gallegos D, Vidgen H (2017). Effectiveness of Australian cooking skill interventions. Br Food J.

[48] Velardo S (2015). The nuances of health literacy, nutrition literacy, and food literacy. J Nutr Educ Behav.

[49] Caruso R, Magon A, Baroni I, Dellafiore F, Arrigoni C, Pittella F, Ausili D (2018). Health literacy in type 2 diabetes patients: a systematic review of systematic reviews. Acta Diabetol.

[50] Leon N, Namadingo H, Bobrow K, Cooper S, Crampin A, Pauly B, Levitt N, Farmer A (2021). Intervention development of a brief messaging intervention for a randomised controlled trial to improve diabetes treatment adherence in sub-Saharan Africa. BMC Public Health.

[51] Bartlett YK, Farmer A, Rea R, French DP (2020). Use of brief messages based on behavior change techniques to encourage medication adherence in people with type 2 diabetes: developmental studies. J Med Internet Res.

[52] Nelson LA, Mayberry LS, Wallston K, Kripalani S, Bergner EM, Osborn CY (2016). Development and usability of REACH: a tailored theory-based text messaging intervention for disadvantaged adults with type 2 diabetes. JMIR Hum Factors.

[53] MacPherson MM, Cranston KD, Locke SR, Bourne JE, Jung ME (2021). Using the Behavior Change Wheel to develop text messages to promote diet and physical activity adherence following a diabetes prevention program. Transl Behav Med.

[54] Hill J (2014). Small steps, big changes. Independent Nurse.

[55] García-Pérez L, Ramallo-Fariña Y, Vallejo-Torres L, Rodríguez-Rodríguez L, González-Pacheco H, Santos-Hernández B, García-Bello MA, Wägner AM, Carmona M, Serrano-Aguilar PG, INDICA team (2022). Cost-effectiveness of multicomponent interventions in type 2 diabetes mellitus in a cluster randomised controlled trial: the INDICA study. BMJ Open.

[56] Lugones-Sánchez C, Recio-Rodríguez JI, Menéndez-Suárez M, Saz-Lara A, Ramirez-Manent JI, Sánchez-Calavera MA, Gómez-Sánchez L, Rodríguez-Sánchez E, García-Ortiz L (2022). Effect of a multicomponent mHealth intervention on the composition of diet in a population with overweight and obesity—randomized clinical trial EVIDENT 3. Nutrients.

[57] Buchanan H, Newton JT, Baker SR, Asimakopoulou K (2021). Adopting the COM-B model and TDF framework in oral and dental research: a narrative review. Community Dent Oral Epidemiol.

[58] Baranowski T, Cullen KW, Nicklas T, Thompson D, Baranowski J (2003). Are current health behavioral change models helpful in guiding prevention of weight gain efforts?. Obes Res.

[59] Abraham C, Michie S (2008). A taxonomy of behavior change techniques used in interventions. Health Psychol.

[60] Webb TL, Joseph J, Yardley L, Michie S (2010). Using the internet to promote health behavior change: a systematic review and meta-analysis of the impact of theoretical basis, use of behavior change techniques, and mode of delivery on efficacy. J Med Internet Res.

[61] Richardson M, Khouja CL, Sutcliffe K, Thomas J (2019). Using the theoretical domains framework and the behavioural change wheel in an overarching synthesis of systematic reviews. BMJ Open.

